# A Curious Case of Snake Venom Addiction as an Alcohol De-Addiction Tool: Pain for Gain?

**DOI:** 10.7759/cureus.19996

**Published:** 2021-11-29

**Authors:** Dhruv Talwar, Sunil Kumar, Sourya Acharya, Gaurav S Jagtap, Sanyukta Hepat

**Affiliations:** 1 Department of Medicine, Jawaharlal Nehru Medical College, Datta Meghe Institute of Medical Sciences, Wardha, IND

**Keywords:** psychoactive fauna, alcohol, addiction, snake bite, snake venom

## Abstract

With the emergence of a wide variety of psychoactive substances which are readily available for abuse, there has been an unfolding of surprising events of addiction to astonishing substances and unusual practices. Snake venom addiction is one such shocking practice that is witnessed throughout the world; however, it is underreported and not frequently talked about. We report one such case from rural central India where a 28-year-old male snake charmer by occupation presented to the emergency department following intoxication with alcohol and snake venom orally. Upon investigation, it turned out to be a case of snake venom addiction which was started by the patient in order to decrease his alcohol addiction through snake bites initially followed by practice of snake venom ingestion.

## Introduction

Substance addiction is a dysfunction of the brain which is chronic and is characterized by a recurring desire to continue taking the substance despite it's harmful consequence [1]. It involves substance-related patterns in excess, which is often socially unacceptable. It is sometimes not the free will of the addict which decides the substance the individual gets addicted to. Various cultural and social aspects influence the substance which is involved in addiction. Snake venom addiction is a practice that has been reported before from different parts of the world, with most of the events involving patients with psychiatric illnesses [2].

Out of the various types of psychoactive substances throughout the globe, snake venom is the most dangerous substance. Animals whose parts, excreted products, or secretions might be of use as a psychoactive agent are known as psychoactive fauna [3]. Psychonaut is the term that is used to denote an individual who tries to use animal parts or products in order to obtain psychoactive benefits [3]. The use of psychoactive fauna involves the superstitious and spiritual beliefs of various groups throughout the world.

Even though the practice of snake venom addiction is relatively common in the Indian subcontinent, there are very few cases reported in the scientific literature [4]. The addicts usually get access to the venom through snake charmers or tribesmen. Different types of dens are made where the addicts are allowed to sit on chairs in order to get a snake bite. The most commonly used snakes by the addicts are Naja naja (cobra), Bungarus caeruleus (common krait) and Opheodrys vernalis (green snake) [2]. Few cases of addiction with semi-poisonous snakes such as rat snakes and green vine have also been reported [5].

The person who is in charge of the snake, holds the snake’s head near the margin of the lip. A sharp tap with a blunt instrument is made on the head of the snake, which makes it bite. Initially, the bites are made in the index finger or little toe, followed by lip, tongue and ear lobes.The addicts usually report a feeling of pricking, which lasts for a duration of 10-40 seconds, followed by a sense of euphoria, muscular weakness and sedation [6].

In a report from Rachi, India, two individuals with multiple substance abuse disorders resorted to the bites of snakes in order to obtain the high sensation or kick, which the substances seemed to lack after chronic use [7].

We report one such case of a 28-year-old male who presented to a rural tertiary care hospital in central India in a state of alcohol intoxication with a history of snake venom consumption.

## Case presentation

A 28-year-old male was brought to the emergency department in a state of irritability following the history of ingestion of alcohol with snake venom, as narrated by his relative, one hour back. The patient was a snake charmer by occupation. The patient had a history of alcohol addiction for 15 years and had a history of repeated failed attempts to quit alcohol addiction in the past. On examination, pulse was 88 beats per minute, regular in rhythm and volume, having no special character, blood pressure was 120/70 mm of Hg and there were no signs of any bleeding tendencies. The patient was conscious and irritable, deep tendon reflexes were normal, bilateral plantar were flexor and there was no neuro deficit.

The abdomen was soft and non-tender, heart sounds were normal, the chest was bilaterally clear.

The patient’s whole blood clotting time was normal, random blood sugar was 80 mg/dl and the rest of the laboratory investigations including complete blood count, renal function test and liver function test were within normal limits. Upon providing injectable thiamine and 25% dextrose 100 ml, the patient improved clinically and became conscious and oriented. The patient then provided a history of snake venom addiction which began four months back, when he got divorced from his wife. It started as an addiction to snake bites which provided him the high sensation that he was using as an alternative to alcohol to reduce his alcohol consumption. Following a single snake bite, the patient used to get a high sensation for a period of 6-7 days, as a result of which his alcohol consumption reduced significantly. For the last one month, the patient was mixing his alcohol drinks with the venom of snakes in order to increase the duration of high effect with less amount alcohol. Cut‐down, Annoyed, Guilt, Eye‐opener (CAGE) score of the patient was calculated to be 3 out of 4 and the alcohol use disorder identifying test score was 26. The patient was started with cognitive behavioral therapy, chlordiazepoxide 10 mg hs (hora somni), along with a short serotonin reuptake inhibitor (escitalopram 10 mg once in a day). He improved clinically and was discharged after one week of admission in stable condition. He is currently doing well on follow-up with the de-addiction team of our hospital, participating actively in group therapies and motivational enhancement therapy.

## Discussion

Nicotine has a wide range of effects on the central nervous system, which are exerted through the nicotinic acetylcholine receptors distributed throughout the brain and predominantly in the hippocampus [8]. One of the subclasses of acetylcholine receptors that is responsible for binding to the toxin α-bungarotoxin of snakes. This toxin exerts its effect by increasing the permeability of calcium in the event of the synapse [9]. The binding of alpha-bungarotoxin is displaced through by-tubocurarine. There is also evidence that another alpha neurotoxin which is released by snakes (elapid), can lead to analgesic effects due to their action on nicotinic acetylcholine receptors and may even be potent enough to replace morphine and can be used in opioid withdrawal [10]. Nicotinic acetylcholine receptors provide rewarding or euphoric experiences, which are usually explored by substance abusers through the activation of the mesolimbic dopaminergic system. It is reasonable to conclude that the experience which the addicts of snake venom describe are exerted through the same pathway [7]. In chronic nicotine addicts, there is upregulation of these nicotinic receptors in the brain, neuroadaptation to nicotine role of the mesocorticolimbic neuroanatomical pathway and desensitization mechanisms which may produce the withdrawal symptoms in such addicts. Snake venom has anti-nociceptive and analgesic property whereas certain neurotoxins have been shown to produce significant analgesia in some animals. The venom of a snake, upon entering the body, leads to the release of bradykinin, peptides, prostaglandins,lysophosphatides and a wide variety of other substances which react slowly.

Snake venom also exerts an effect on muscarinic receptors, which play a vital role in physiological processes like memory enhancement and learning. Selective antagonist to M4 receptor, which is known as MT3, can be extracted from snake venom of mamba snake can act on the hippocampus to enhance the retrieval of memory.

Snake venom addiction is a dangerous practice with snake bite being a potentially lethal event with varied presentations usually requiring antisnake venom and neostigmine playing an essential role in the treatment [11,12]. However, there have been reported cases where patients with deadly snakebites from snakes such as Bangarus Caeru recovered completely without the need for any treatment owing to the antibodies made from previous snake bites [13]. This can explain why the addicts do not face lethal complications from snake bites as repeated snake bites with the injection of small doses of snake venom induces immunity against snake venom intoxication in the future. Such a phenomenon was seen in a reptile handler who was bitten by king cobra Ophiophagus hannah and developed antibodies against its venom [14].

Very few cases of snake venom addiction have been reported from India. In a case report from Tamil Nadu, two software engineers were reported to be addicted to snake venom with no prior psychiatric illness. They reported having a high sensation due to snake venom along with an increase in sexual desire [5]. Another case series from Mumbai, India reported three cases of snake venom addicts who used to procure the venom through rave parties [15].A report from Mangalore, India described an 18-year-old male who used to get a snake bite on the tongue after which felt an increased sense of well-being, blurry vision and sedation. He would then sleep for a duration of thirty to thirty-six hours and later did not report having any craving or psychiatric manifestation [16].

Our patient had different social and cultural influences that drove him to the road of snake venom addiction. He was an alcoholic with repeated failed attempts of quitting alcohol consumption. He was a snake charmer by occupation with easy access to snakes and snake venom. Also, he had a recent history of a disturbing event of a divorce from his wife, which had probably resulted from his addiction to alcohol. He belonged to a village from the rural parts of central India and probably using snake venom as a stronger and yet easily accessible and cheaper alternative appealed to him. Various superstitious beliefs regarding a snake in rural India might have also influenced him into using snake venom as an alternative to alcohol to achieve the high sensations he earlier used to get from alcohol.

As our patient was a chronic abuser and was dependent on alcohol and snake venom, a tailored prolonged individual behavioral therapy and motivational enhancement therapy helped him recover along with serotonin short reuptake inhibitor.

## Conclusions

We conclude that a dangerous addiction such as snake venom might result from an attempt to counter the addiction of alcohol which is molded by religious, cultural and spiritual beliefs of the society along with underlying psychiatric illness such as depression or anxiety. Hence. the practitioners should have snake venom addiction as their differential when working in a vicinity where snake bite addiction might develop due to socio-cultural or religious factors.

## References

[1] Zou Z, Wang H, d'Oleire Uquillas F, Wang X, Ding J, Chen H (2017). Definition of substance and non-substance addiction. Substance and Non-substance Addiction.

[2] Das S, Barnwal P, Maiti T, Ramasamy A, Mondal S, Babu D (2017). Addiction to snake venom. Subst Use Misuse.

[3] Prince JD (2011). A dictionary of hallucinations. J Med Libr Assoc.

[4] Shukla L, Reddy SS, Kandasamy A, Benegal V (2017). What kills everyone, gives a high for some-recreational snake envenomation. Asian J Psychiatr.

[5] Senthilkumaran S, Shah S, Balamurugan N, Menezes RG, Thirumalaikolundusubramanian P (2013). Repeated snake bite for recreation: mechanisms and implications. Int J Crit Illn Inj Sci.

[6] Kautilya V, Bhodka P (2012). Snake venom - the new rage to get high. J Indian Soc Toxicol.

[7] Katshu MZ, Dubey I, Khess CR, Sarkhel S (2011). Snake bite as a novel form of substance abuse: personality profiles and cultural perspectives. Subst Abus.

[8] Freedman R, Wetmore C, Strömberg I, Leonard S, Olson L (1993). Alpha-bungarotoxin binding to hippocampal interneurons: immunocytochemical characterization and effects on growth factor expression. J Neurosci.

[9] Koh DC, Armugam A, Jeyaseelan K (2006). Snake venom components and their applications in biomedicine. Cell Mol Life Sci.

[10] Chen ZX, Zhang HL, Gu ZL (2006). A long-form α-neurotoxin from cobra venom produces potent opioid-independent analgesia. Acta Pharmacol Sin.

[11] Irshad VS, Godhiwala P, Kumar S, Bagga CS, Gupta A (2021). Role of neostigmine in neurotoxic snake bite. J Evolution Med Dent Sci.

[12] Godhiwala P, Acharya S, Andhale A, Shukla S, Kumar S (2021). Snake bite presenting as haemolytic uremic syndrome: a rare case report. J Clin Diagn Res.

[13] Haast WE, Winer ML (1955). Complete and spontaneous recovery from the bite of a blue krait snake (Bungarus caeruleiis). Am J Trop Med Hyg.

[14] Tun-Pe Tun-Pe, Aye-Aye-Myint Aye-Aye-Myint, Warrell DA, Tin-Myint Tin-Myint (1995). King cobra (Ophiophagus hannah) bites in Myanmar: venom antigen levels and development of venom antibodies. Toxicon.

[15] Umate MK, Khot P, Salukhe R, Kale V (2015). Snake venom abuse in rave parties: a case report. Indian J Ment Health.

[16] Krishnamurthy K, Braganza WJ (2013). A unique case of substance abuse. Muller J Med Sci Res.

